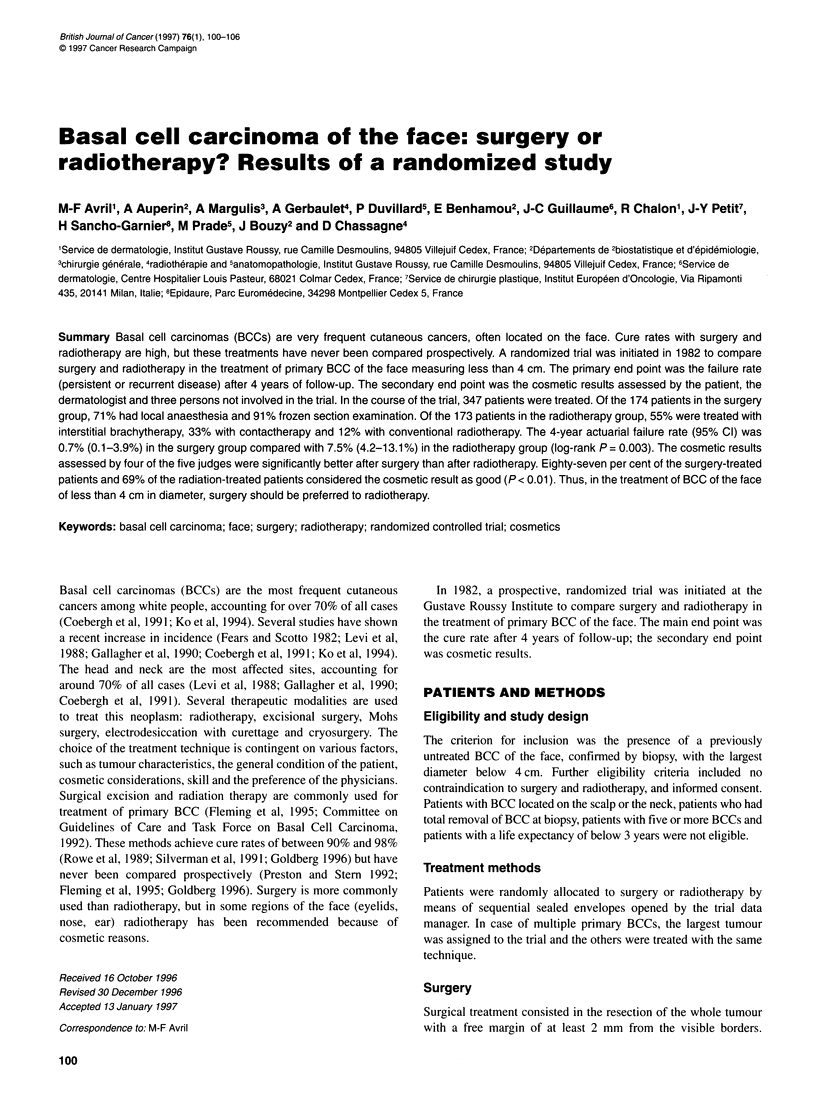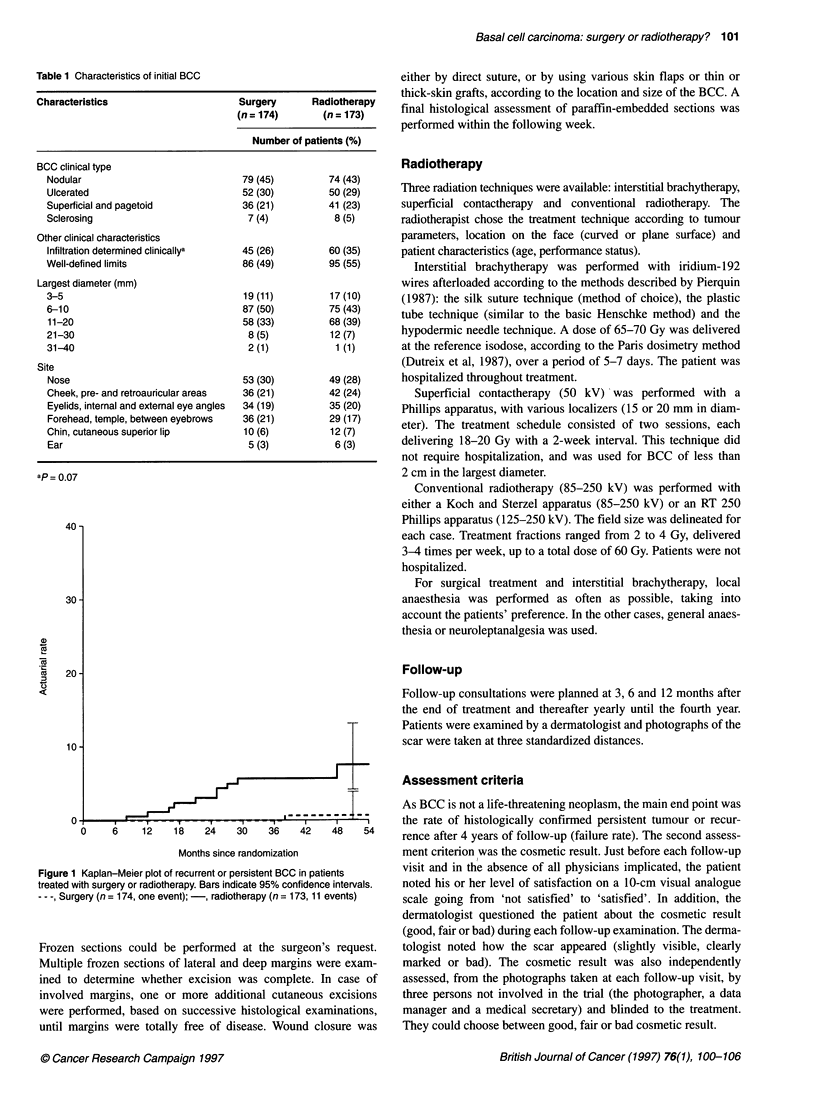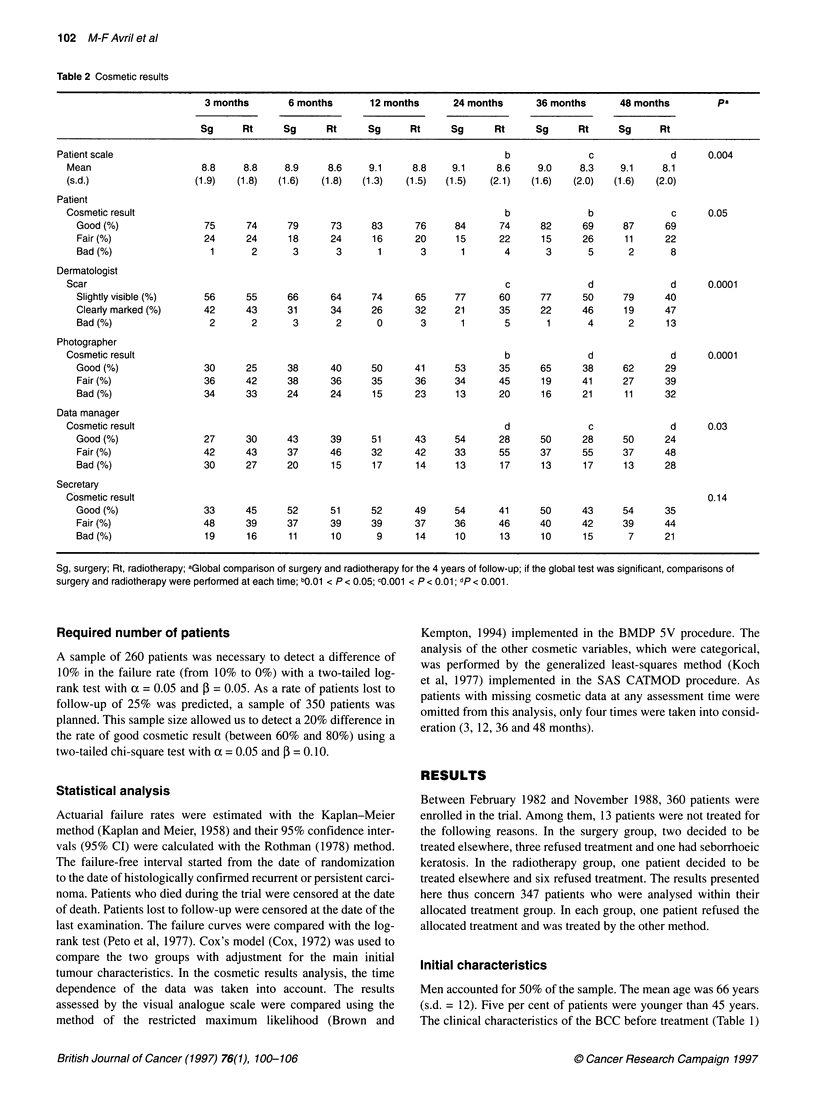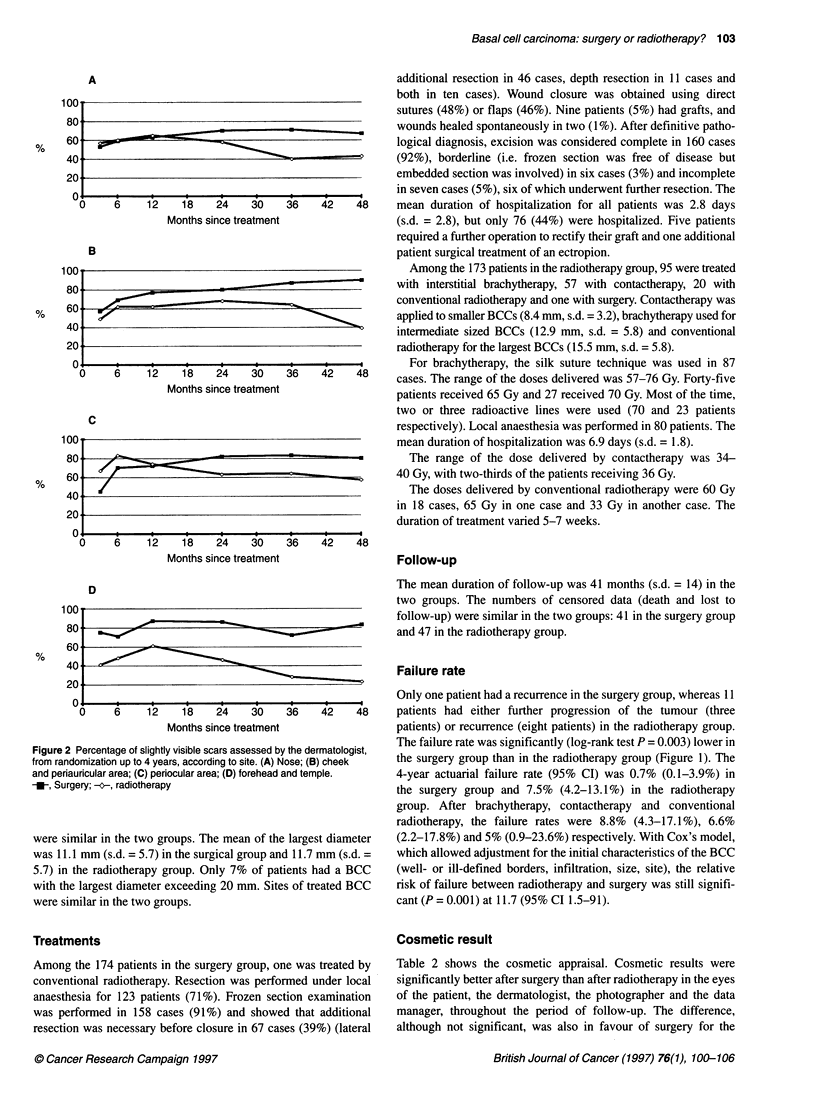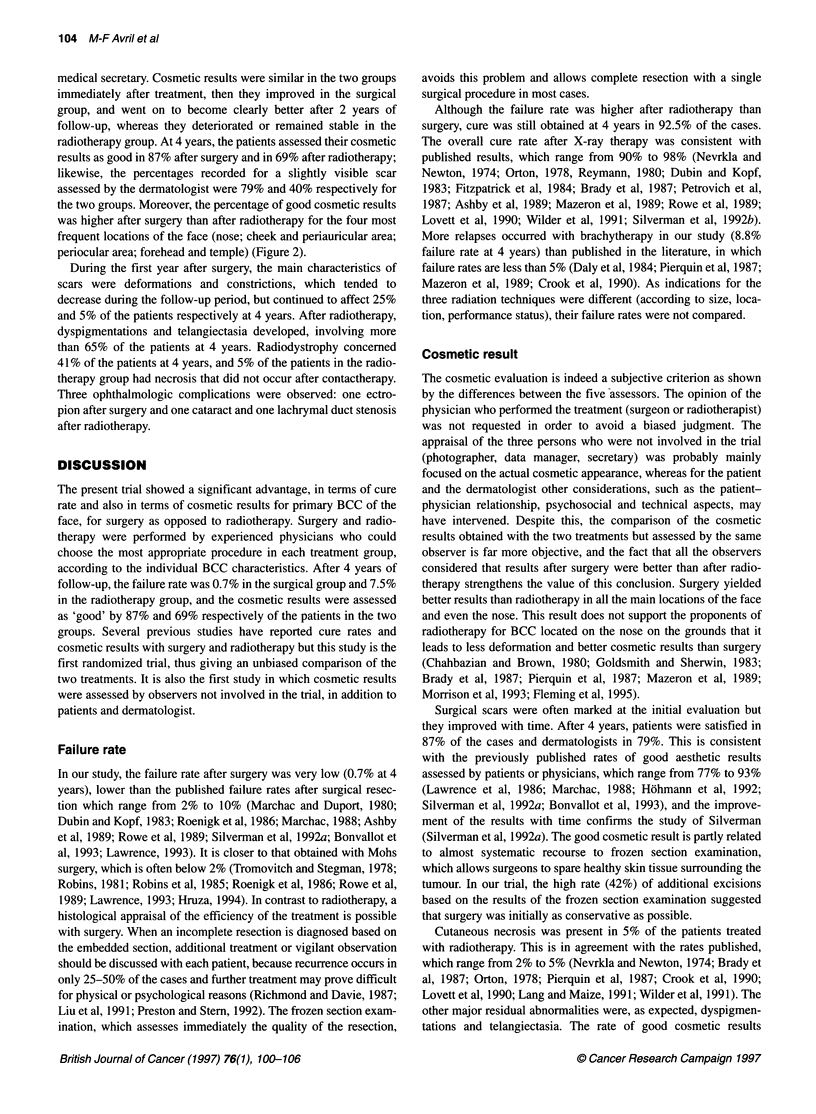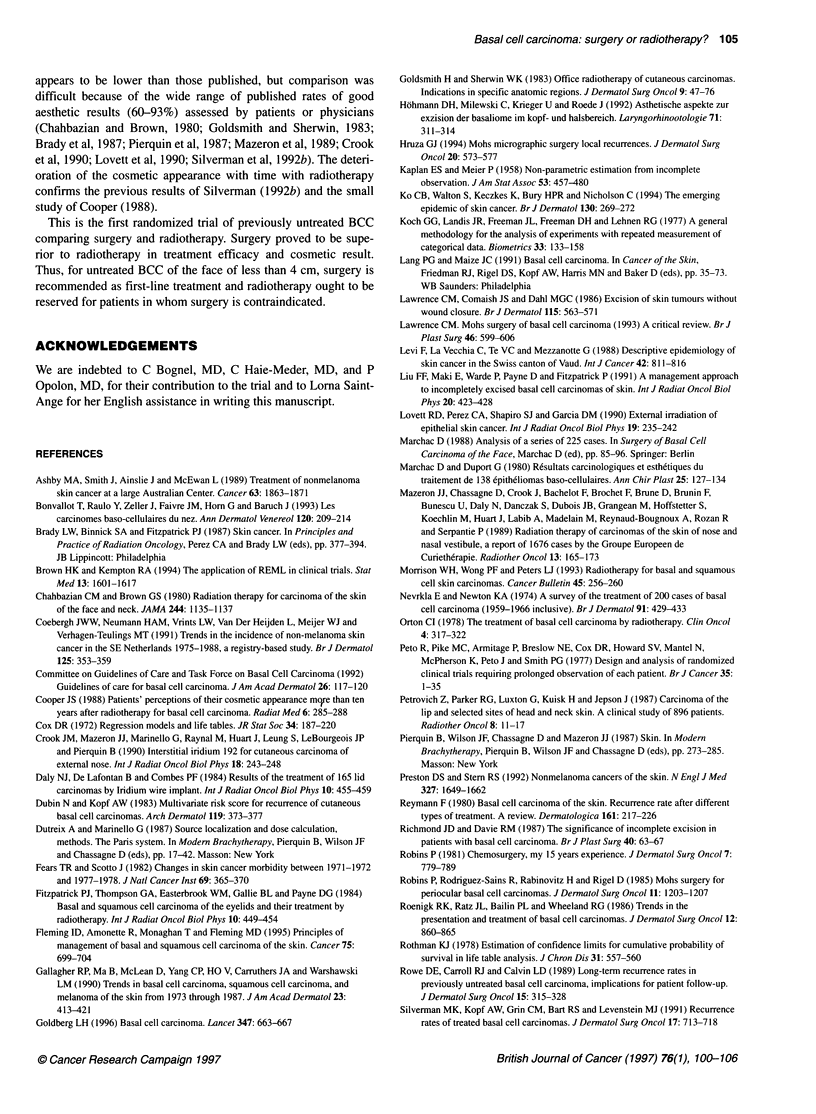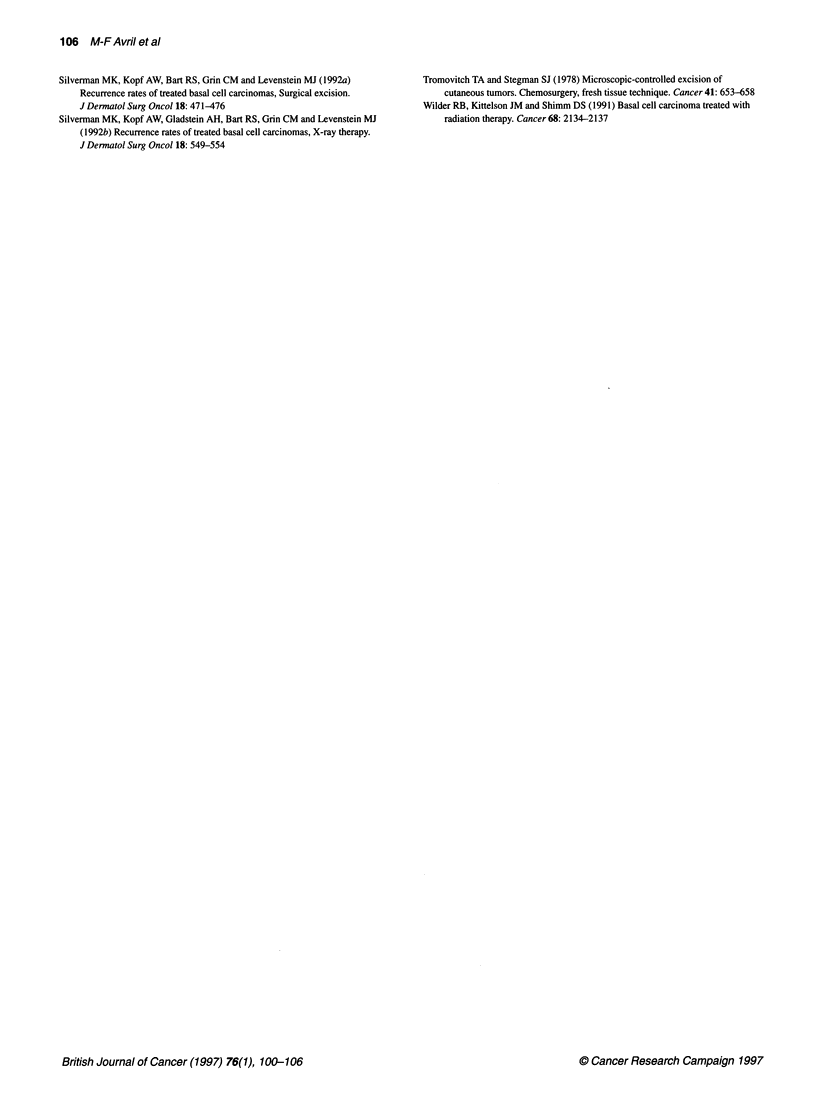# Basal cell carcinoma of the face: surgery or radiotherapy? Results of a randomized study.

**DOI:** 10.1038/bjc.1997.343

**Published:** 1997

**Authors:** M. F. Avril, A. Auperin, A. Margulis, A. Gerbaulet, P. Duvillard, E. Benhamou, J. C. Guillaume, R. Chalon, J. Y. Petit, H. Sancho-Garnier, M. Prade, J. Bouzy, D. Chassagne

**Affiliations:** Service de dermatologie, Institut Gustave Roussy, Villejuif, France.

## Abstract

Basal cell carcinomas (BCCs) are very frequent cutaneous cancers, often located on the face. Cure rates with surgery and radiotherapy are high, but these treatments have never been compared prospectively. A randomized trial was initiated in 1982 to compare surgery and radiotherapy in the treatment of primary BCC of the face measuring less than 4 cm. The primary end point was the failure rate (persistent or recurrent disease) after 4 years of follow-up. The secondary end point was the cosmetic results assessed by the patient, the dermatologist and three persons not involved in the trial. In the course of the trial, 347 patients were treated. Of the 174 patients in the surgery group, 71% had local anaesthesia and 91% frozen section examination. Of the 173 patients in the radiotherapy group, 55% were treated with interstitial brachytherapy, 33% with contactherapy and 12% with conventional radiotherapy. The 4-year actuarial failure rate (95% CI) was 0.7% (0.1-3.9%) in the surgery group compared with 7.5% (4.2-13.1%) in the radiotherapy group (log-rank P = 0.003). The cosmetic results assessed by four of the five judges were significantly better after surgery than after radiotherapy. Eighty-seven per cent of the surgery-treated patients and 69% of the radiation-treated patients considered the cosmetic result as good (P < 0.01). Thus, in the treatment of BCC of the face of less than 4 cm in diameter, surgery should be preferred to radiotherapy.


*British Journal of Cancer* (1997) **76**(1), 100–106
© 1997 Cancer Research Campaign

### Basal cell carcinoma of the face: surgery or radiotherapy? Results of a randomized study

**M-F Avril[1], A Auperin[2], A Margulis[3], A Gerbaulet[4], P Duvillard[5], E Benhamou[2], J-C Guillaume[6], R Chalon[1], J-Y Petit[7], H Sancho-Garnier[8], M Prade[5], J Bouzy[2] and D Chassagne[4]**

[1]Service de dermatologie, Institut Gustave Roussy, rue Camille Desmoulins, 94805 Villejuif Cedex, France; [2]Départements de [2]biostatistique et d'épidémiologie, [3]chirurgie générale, [4]radiothérapie and [5]anatomopathologie, Institut Gustave Roussy, rue Camille Desmoulins, 94805 Villejuif Cedex, France; [6]Service de dermatologie, Centre Hospitalier Louis Pasteur, 68021 Colmar Cedex, France; [7]Service de chirurgie plastique, Institut Européen d'Oncologie, Via Ripamonti 435, 20141 Milan, Italie; [8]Epidaure, Parc Euromédecine, 34298 Montpellier Cedex 5, France

**Summary** Basal cell carcinomas (BCCs) are very frequent cutaneous cancers, often located on the face. Cure rates with surgery and radiotherapy are high, but these treatments have never been compared prospectively. A randomized trial was initiated in 1982 to compare surgery and radiotherapy in the treatment of primary BCC of the face measuring less than 4 cm. The primary end point was the failure rate (persistent or recurrent disease) after 4 years of follow-up. The secondary end point was the cosmetic results assessed by the patient, the dermatologist and three persons not involved in the trial. In the course of the trial, 347 patients were treated. Of the 174 patients in the surgery group, 71% had local anaesthesia and 91% frozen section examination. Of the 173 patients in the radiotherapy group, 55% were treated with interstitial brachytherapy, 33% with contactherapy and 12% with conventional radiotherapy. The 4-year actuarial failure rate (95% CI) was 0.7% (0.1–3.9%) in the surgery group compared with 7.5% (4.2–13.1%) in the radiotherapy group (log-rank $P = 0.003$). The cosmetic results assessed by four of the five judges were significantly better after surgery than after radiotherapy. Eighty-seven per cent of the surgery-treated patients and 69% of the radiation-treated patients considered the cosmetic result as good ($P < 0.01$). Thus, in the treatment of BCC of the face of less than 4 cm in diameter, surgery should be preferred to radiotherapy.

**Keywords:** basal cell carcinoma; face; surgery; radiotherapy; randomized controlled trial; cosmetics

Basal cell carcinomas (BCCs) are the most frequent cutaneous cancers among white people, accounting for over 70% of all cases (Coebergh et al, 1991; Ko et al, 1994). Several studies have shown a recent increase in incidence (Fears and Scotto 1982; Levi et al, 1988; Gallagher et al, 1990; Coebergh et al, 1991; Ko et al, 1994). The head and neck are the most affected sites, accounting for around 70% of all cases (Levi et al, 1988; Gallagher et al, 1990; Coebergh et al, 1991). Several therapeutic modalities are used to treat this neoplasm: radiotherapy, excisional surgery, Mohs surgery, electrodesiccation with curettage and cryosurgery. The choice of the treatment technique is contingent on various factors, such as tumour characteristics, the general condition of the patient, cosmetic considerations, skill and the preference of the physicians. Surgical excision and radiation therapy are commonly used for treatment of primary BCC (Fleming et al, 1995; Committee on Guidelines of Care and Task Force on Basal Cell Carcinoma, 1992). These methods achieve cure rates of between 90% and 98% (Rowe et al, 1989; Silverman et al, 1991; Goldberg 1996) but have never been compared prospectively (Preston and Stern 1992; Fleming et al, 1995; Goldberg 1996). Surgery is more commonly used than radiotherapy, but in some regions of the face (eyelids, nose, ear) radiotherapy has been recommended because of cosmetic reasons.

*Received 16 October 1996*
*Revised 30 December 1996*
*Accepted 13 January 1997*

*Correspondence to:* M-F Avril

In 1982, a prospective, randomized trial was initiated at the Gustave Roussy Institute to compare surgery and radiotherapy in the treatment of primary BCC of the face. The main end point was the cure rate after 4 years of follow-up; the secondary end point was cosmetic results.

### PATIENTS AND METHODS

### Eligibility and study design

The criterion for inclusion was the presence of a previously untreated BCC of the face, confirmed by biopsy, with the largest diameter below 4 cm. Further eligibility criteria included no contraindication to surgery and radiotherapy, and informed consent. Patients with BCC located on the scalp or the neck, patients who had total removal of BCC at biopsy, patients with five or more BCCs and patients with a life expectancy of below 3 years were not eligible.

### Treatment methods

Patients were randomly allocated to surgery or radiotherapy by means of sequential sealed envelopes opened by the trial data manager. In case of multiple primary BCCs, the largest tumour was assigned to the trial and the others were treated with the same technique.

### Surgery

Surgical treatment consisted in the resection of the whole tumour with a free margin of at least 2 mm from the visible borders.

**Table 1** Characteristics of initial BCC

| Characteristics | Surgery ($n$ = 174) | Radiotherapy ($n$ = 173) |
|---|---|---|
| | Number of patients (%) | |
| BCC clinical type | | |
| Nodular | 79 (45) | 74 (43) |
| Ulcerated | 52 (30) | 50 (29) |
| Superficial and pagetoid | 36 (21) | 41 (23) |
| Sclerosing | 7 (4) | 8 (5) |
| Other clinical characteristics | | |
| Infiltration determined clinically[a] | 45 (26) | 60 (35) |
| Well-defined limits | 86 (49) | 95 (55) |
| Largest diameter (mm) | | |
| 3–5 | 19 (11) | 17 (10) |
| 6–10 | 87 (50) | 75 (43) |
| 11–20 | 58 (33) | 68 (39) |
| 21–30 | 8 (5) | 12 (7) |
| 31–40 | 2 (1) | 1 (1) |
| Site | | |
| Nose | 53 (30) | 49 (28) |
| Cheek, pre- and retroauricular areas | 36 (21) | 42 (24) |
| Eyelids, internal and external eye angles | 34 (19) | 35 (20) |
| Forehead, temple, between eyebrows | 36 (21) | 29 (17) |
| Chin, cutaneous superior lip | 10 (6) | 12 (7) |
| Ear | 5 (3) | 6 (3) |

[a]$P$ = 0.07

**Figure 1** Kaplan–Meier plot of recurrent or persistent BCC in patients treated with surgery or radiotherapy. Bars indicate 95% confidence intervals. - - -, Surgery ($n$ = 174, one event); —, radiotherapy ($n$ = 173, 11 events)

Frozen sections could be performed at the surgeon's request. Multiple frozen sections of lateral and deep margins were examined to determine whether excision was complete. In case of involved margins, one or more additional cutaneous excisions were performed, based on successive histological examinations, until margins were totally free of disease. Wound closure was either by direct suture, or by using various skin flaps or thin or thick-skin grafts, according to the location and size of the BCC. A final histological assessment of paraffin-embedded sections was performed within the following week.

### Radiotherapy

Three radiation techniques were available: interstitial brachytherapy, superficial contactherapy and conventional radiotherapy. The radiotherapist chose the treatment technique according to tumour parameters, location on the face (curved or plane surface) and patient characteristics (age, performance status).

Interstitial brachytherapy was performed with iridium-192 wires afterloaded according to the methods described by Pierquin (1987): the silk suture technique (method of choice), the plastic tube technique (similar to the basic Henschke method) and the hypodermic needle technique. A dose of 65–70 Gy was delivered at the reference isodose, according to the Paris dosimetry method (Dutreix et al, 1987), over a period of 5–7 days. The patient was hospitalized throughout treatment.

Superficial contactherapy (50 kV) was performed with a Phillips apparatus, with various localizers (15 or 20 mm in diameter). The treatment schedule consisted of two sessions, each delivering 18–20 Gy with a 2-week interval. This technique did not require hospitalization, and was used for BCC of less than 2 cm in the largest diameter.

Conventional radiotherapy (85–250 kV) was performed with either a Koch and Sterzel apparatus (85–250 kV) or an RT 250 Phillips apparatus (125–250 kV). The field size was delineated for each case. Treatment fractions ranged from 2 to 4 Gy, delivered 3–4 times per week, up to a total dose of 60 Gy. Patients were not hospitalized.

For surgical treatment and interstitial brachytherapy, local anaesthesia was performed as often as possible, taking into account the patients' preference. In the other cases, general anaesthesia or neuroleptanalgesia was used.

### Follow-up

Follow-up consultations were planned at 3, 6 and 12 months after the end of treatment and thereafter yearly until the fourth year. Patients were examined by a dermatologist and photographs of the scar were taken at three standardized distances.

### Assessment criteria

As BCC is not a life-threatening neoplasm, the main end point was the rate of histologically confirmed persistent tumour or recurrence after 4 years of follow-up (failure rate). The second assessment criterion was the cosmetic result. Just before each follow-up visit and in the absence of all physicians implicated, the patient noted his or her level of satisfaction on a 10-cm visual analogue scale going from 'not satisfied' to 'satisfied'. In addition, the dermatologist questioned the patient about the cosmetic result (good, fair or bad) during each follow-up examination. The dermatologist noted how the scar appeared (slightly visible, clearly marked or bad). The cosmetic result was also independently assessed, from the photographs taken at each follow-up visit, by three persons not involved in the trial (the photographer, a data manager and a medical secretary) and blinded to the treatment. They could choose between good, fair or bad cosmetic result.

© Cancer Research Campaign 1997

**Table 2** Cosmetic results

| | 3 months | | 6 months | | 12 months | | 24 months | | 36 months | | 48 months | | $P^a$ |
|---|---|---|---|---|---|---|---|---|---|---|---|---|---|
| | Sg | Rt | Sg | Rt | Sg | Rt | Sg | Rt | Sg | Rt | Sg | Rt | |
| Patient scale | | | | | | | | b | | c | | d | 0.004 |
| Mean | 8.8 | 8.8 | 8.9 | 8.6 | 9.1 | 8.8 | 9.1 | 8.6 | 9.0 | 8.3 | 9.1 | 8.1 | |
| (s.d.) | (1.9) | (1.8) | (1.6) | (1.8) | (1.3) | (1.5) | (1.5) | (2.1) | (1.6) | (2.0) | (1.6) | (2.0) | |
| Patient | | | | | | | | | | | | | |
| Cosmetic result | | | | | | | | b | | b | | c | 0.05 |
| Good (%) | 75 | 74 | 79 | 73 | 83 | 76 | 84 | 74 | 82 | 69 | 87 | 69 | |
| Fair (%) | 24 | 24 | 18 | 24 | 16 | 20 | 15 | 22 | 15 | 26 | 11 | 22 | |
| Bad (%) | 1 | 2 | 3 | 3 | 1 | 3 | 1 | 4 | 3 | 5 | 2 | 8 | |
| Dermatologist | | | | | | | | | | | | | |
| Scar | | | | | | | | c | | d | | d | 0.0001 |
| Slightly visible (%) | 56 | 55 | 66 | 64 | 74 | 65 | 77 | 60 | 77 | 50 | 79 | 40 | |
| Clearly marked (%) | 42 | 43 | 31 | 34 | 26 | 32 | 21 | 35 | 22 | 46 | 19 | 47 | |
| Bad (%) | 2 | 2 | 3 | 2 | 0 | 3 | 1 | 5 | 1 | 4 | 2 | 13 | |
| Photographer | | | | | | | | | | | | | |
| Cosmetic result | | | | | | | | b | | d | | d | 0.0001 |
| Good (%) | 30 | 25 | 38 | 40 | 50 | 41 | 53 | 35 | 65 | 38 | 62 | 29 | |
| Fair (%) | 36 | 42 | 38 | 36 | 35 | 36 | 34 | 45 | 19 | 41 | 27 | 39 | |
| Bad (%) | 34 | 33 | 24 | 24 | 15 | 23 | 13 | 20 | 16 | 21 | 11 | 32 | |
| Data manager | | | | | | | | | | | | | |
| Cosmetic result | | | | | | | | d | | c | | d | 0.03 |
| Good (%) | 27 | 30 | 43 | 39 | 51 | 43 | 54 | 28 | 50 | 28 | 50 | 24 | |
| Fair (%) | 42 | 43 | 37 | 46 | 32 | 42 | 33 | 55 | 37 | 55 | 37 | 48 | |
| Bad (%) | 30 | 27 | 20 | 15 | 17 | 14 | 13 | 17 | 13 | 17 | 13 | 28 | |
| Secretary | | | | | | | | | | | | | |
| Cosmetic result | | | | | | | | | | | | | 0.14 |
| Good (%) | 33 | 45 | 52 | 51 | 52 | 49 | 54 | 41 | 50 | 43 | 54 | 35 | |
| Fair (%) | 48 | 39 | 37 | 39 | 39 | 37 | 36 | 46 | 40 | 42 | 39 | 44 | |
| Bad (%) | 19 | 16 | 11 | 10 | 9 | 14 | 10 | 13 | 10 | 15 | 7 | 21 | |

Sg, surgery; Rt, radiotherapy; [a]Global comparison of surgery and radiotherapy for the 4 years of follow-up; if the global test was significant, comparisons of surgery and radiotherapy were performed at each time; [b]$0.01 < P < 0.05$; [c]$0.001 < P < 0.01$; [d]$P < 0.001$.

### Required number of patients

A sample of 260 patients was necessary to detect a difference of 10% in the failure rate (from 10% to 0%) with a two-tailed log-rank test with $\alpha = 0.05$ and $\beta = 0.05$. As a rate of patients lost to follow-up of 25% was predicted, a sample of 350 patients was planned. This sample size allowed us to detect a 20% difference in the rate of good cosmetic result (between 60% and 80%) using a two-tailed chi-square test with $\alpha = 0.05$ and $\beta = 0.10$.

### Statistical analysis

Actuarial failure rates were estimated with the Kaplan–Meier method (Kaplan and Meier, 1958) and their 95% confidence intervals (95% CI) were calculated with the Rothman (1978) method. The failure-free interval started from the date of randomization to the date of histologically confirmed recurrent or persistent carcinoma. Patients who died during the trial were censored at the date of death. Patients lost to follow-up were censored at the date of the last examination. The failure curves were compared with the log-rank test (Peto et al, 1977). Cox's model (Cox, 1972) was used to compare the two groups with adjustment for the main initial tumour characteristics. In the cosmetic results analysis, the time dependence of the data was taken into account. The results assessed by the visual analogue scale were compared using the method of the restricted maximum likelihood (Brown and Kempton, 1994) implemented in the BMDP 5V procedure. The analysis of the other cosmetic variables, which were categorical, was performed by the generalized least-squares method (Koch et al, 1977) implemented in the SAS CATMOD procedure. As patients with missing cosmetic data at any assessment time were omitted from this analysis, only four times were taken into consideration (3, 12, 36 and 48 months).

### RESULTS

Between February 1982 and November 1988, 360 patients were enrolled in the trial. Among them, 13 patients were not treated for the following reasons. In the surgery group, two decided to be treated elsewhere, three refused treatment and one had seborrhoeic keratosis. In the radiotherapy group, one patient decided to be treated elsewhere and six refused treatment. The results presented here thus concern 347 patients who were analysed within their allocated treatment group. In each group, one patient refused the allocated treatment and was treated by the other method.

### Initial characteristics

Men accounted for 50% of the sample. The mean age was 66 years (s.d. = 12). Five per cent of patients were younger than 45 years. The clinical characteristics of the BCC before treatment (Table 1)

 © Cancer Research Campaign 1997

**Figure 2** Percentage of slightly visible scars assessed by the dermatologist, from randomization up to 4 years, according to site. (**A**) Nose; (**B**) cheek and periauricular area; (**C**) periocular area; (**D**) forehead and temple. -■-, Surgery; -◇-, radiotherapy were similar in the two groups. The mean of the largest diameter was 11.1 mm (s.d. = 5.7) in the surgical group and 11.7 mm (s.d. = 5.7) in the radiotherapy group. Only 7% of patients had a BCC with the largest diameter exceeding 20 mm. Sites of treated BCC were similar in the two groups.

### Treatments

Among the 174 patients in the surgery group, one was treated by conventional radiotherapy. Resection was performed under local anaesthesia for 123 patients (71%). Frozen section examination was performed in 158 cases (91%) and showed that additional resection was necessary before closure in 67 cases (39%) (lateral additional resection in 46 cases, depth resection in 11 cases and both in ten cases). Wound closure was obtained using direct sutures (48%) or flaps (46%). Nine patients (5%) had grafts, and wounds healed spontaneously in two (1%). After definitive pathological diagnosis, excision was considered complete in 160 cases (92%), borderline (i.e. frozen section was free of disease but embedded section was involved) in six cases (3%) and incomplete in seven cases (5%), six of which underwent further resection. The mean duration of hospitalization for all patients was 2.8 days (s.d. = 2.8), but only 76 (44%) were hospitalized. Five patients required a further operation to rectify their graft and one additional patient surgical treatment of an ectropion.

Among the 173 patients in the radiotherapy group, 95 were treated with interstitial brachytherapy, 57 with contactherapy, 20 with conventional radiotherapy and one with surgery. Contactherapy was applied to smaller BCCs (8.4 mm, s.d. = 3.2), brachytherapy used for intermediate sized BCCs (12.9 mm, s.d. = 5.8) and conventional radiotherapy for the largest BCCs (15.5 mm, s.d. = 5.8).

For brachytherapy, the silk suture technique was used in 87 cases. The range of the doses delivered was 57–76 Gy. Forty-five patients received 65 Gy and 27 received 70 Gy. Most of the time, two or three radioactive lines were used (70 and 23 patients respectively). Local anaesthesia was performed in 80 patients. The mean duration of hospitalization was 6.9 days (s.d. = 1.8).

The range of the dose delivered by contactherapy was 34–40 Gy, with two-thirds of the patients receiving 36 Gy.

The doses delivered by conventional radiotherapy were 60 Gy in 18 cases, 65 Gy in one case and 33 Gy in another case. The duration of treatment varied 5–7 weeks.

### Follow-up

The mean duration of follow-up was 41 months (s.d. = 14) in the two groups. The numbers of censored data (death and lost to follow-up) were similar in the two groups: 41 in the surgery group and 47 in the radiotherapy group.

### Failure rate

Only one patient had a recurrence in the surgery group, whereas 11 patients had either further progression of the tumour (three patients) or recurrence (eight patients) in the radiotherapy group. The failure rate was significantly (log-rank test $P = 0.003$) lower in the surgery group than in the radiotherapy group (Figure 1). The 4-year actuarial failure rate (95% CI) was 0.7% (0.1–3.9%) in the surgery group and 7.5% (4.2–13.1%) in the radiotherapy group. After brachytherapy, contactherapy and conventional radiotherapy, the failure rates were 8.8% (4.3–17.1%), 6.6% (2.2–17.8%) and 5% (0.9–23.6%) respectively. With Cox's model, which allowed adjustment for the initial characteristics of the BCC (well- or ill-defined borders, infiltration, size, site), the relative risk of failure between radiotherapy and surgery was still significant ($P = 0.001$) at 11.7 (95% CI 1.5–91).

### Cosmetic result

Table 2 shows the cosmetic appraisal. Cosmetic results were significantly better after surgery than after radiotherapy in the eyes of the patient, the dermatologist, the photographer and the data manager, throughout the period of follow-up. The difference, although not significant, was also in favour of surgery for the

© Cancer Research Campaign 1997 

medical secretary. Cosmetic results were similar in the two groups immediately after treatment, then they improved in the surgical group, and went on to become clearly better after 2 years of follow-up, whereas they deteriorated or remained stable in the radiotherapy group. At 4 years, the patients assessed their cosmetic results as good in 87% after surgery and in 69% after radiotherapy; likewise, the percentages recorded for a slightly visible scar assessed by the dermatologist were 79% and 40% respectively for the two groups. Moreover, the percentage of good cosmetic results was higher after surgery than after radiotherapy for the four most frequent locations of the face (nose; cheek and periauricular area; periocular area; forehead and temple) (Figure 2).

During the first year after surgery, the main characteristics of scars were deformations and constrictions, which tended to decrease during the follow-up period, but continued to affect 25% and 5% of the patients respectively at 4 years. After radiotherapy, dyspigmentations and telangiectasia developed, involving more than 65% of the patients at 4 years. Radiodystrophy concerned 41% of the patients at 4 years, and 5% of the patients in the radiotherapy group had necrosis that did not occur after contactherapy. Three ophthalmologic complications were observed: one ectropion after surgery and one cataract and one lachrymal duct stenosis after radiotherapy.

### DISCUSSION

The present trial showed a significant advantage, in terms of cure rate and also in terms of cosmetic results for primary BCC of the face, for surgery as opposed to radiotherapy. Surgery and radiotherapy were performed by experienced physicians who could choose the most appropriate procedure in each treatment group, according to the individual BCC characteristics. After 4 years of follow-up, the failure rate was 0.7% in the surgical group and 7.5% in the radiotherapy group, and the cosmetic results were assessed as 'good' by 87% and 69% respectively of the patients in the two groups. Several previous studies have reported cure rates and cosmetic results with surgery and radiotherapy but this study is the first randomized trial, thus giving an unbiased comparison of the two treatments. It is also the first study in which cosmetic results were assessed by observers not involved in the trial, in addition to patients and dermatologist.

### Failure rate

In our study, the failure rate after surgery was very low (0.7% at 4 years), lower than the published failure rates after surgical resection which range from 2% to 10% (Marchac and Duport, 1980; Dubin and Kopf, 1983; Roenigk et al, 1986; Marchac, 1988; Ashby et al, 1989; Rowe et al, 1989; Silverman et al, 1992*a*; Bonvallot et al, 1993; Lawrence, 1993). It is closer to that obtained with Mohs surgery, which is often below 2% (Tromovitch and Stegman, 1978; Robins, 1981; Robins et al, 1985; Roenigk et al, 1986; Rowe et al, 1989; Lawrence, 1993; Hruza, 1994). In contrast to radiotherapy, a histological appraisal of the efficiency of the treatment is possible with surgery. When an incomplete resection is diagnosed based on the embedded section, additional treatment or vigilant observation should be discussed with each patient, because recurrence occurs in only 25–50% of the cases and further treatment may prove difficult for physical or psychological reasons (Richmond and Davie, 1987; Liu et al, 1991; Preston and Stern, 1992). The frozen section examination, which assesses immediately the quality of the resection, avoids this problem and allows complete resection with a single surgical procedure in most cases.

Although the failure rate was higher after radiotherapy than surgery, cure was still obtained at 4 years in 92.5% of the cases. The overall cure rate after X-ray therapy was consistent with published results, which range from 90% to 98% (Nevrkla and Newton, 1974; Orton, 1978, Reymann, 1980; Dubin and Kopf, 1983; Fitzpatrick et al, 1984; Brady et al, 1987; Petrovich et al, 1987; Ashby et al, 1989; Mazeron et al, 1989; Rowe et al, 1989; Lovett et al, 1990; Wilder et al, 1991; Silverman et al, 1992*b*). More relapses occurred with brachytherapy in our study (8.8% failure rate at 4 years) than published in the literature, in which failure rates are less than 5% (Daly et al, 1984; Pierquin et al, 1987; Mazeron et al, 1989; Crook et al, 1990). As indications for the three radiation techniques were different (according to size, location, performance status), their failure rates were not compared.

### Cosmetic result

The cosmetic evaluation is indeed a subjective criterion as shown by the differences between the five assessors. The opinion of the physician who performed the treatment (surgeon or radiotherapist) was not requested in order to avoid a biased judgment. The appraisal of the three persons who were not involved in the trial (photographer, data manager, secretary) was probably mainly focused on the actual cosmetic appearance, whereas for the patient and the dermatologist other considerations, such as the patient–physician relationship, psychosocial and technical aspects, may have intervened. Despite this, the comparison of the cosmetic results obtained with the two treatments but assessed by the same observer is far more objective, and the fact that all the observers considered that results after surgery were better than after radiotherapy strengthens the value of this conclusion. Surgery yielded better results than radiotherapy in all the main locations of the face and even the nose. This result does not support the proponents of radiotherapy for BCC located on the nose on the grounds that it leads to less deformation and better cosmetic results than surgery (Chahbazian and Brown, 1980; Goldsmith and Sherwin, 1983; Brady et al, 1987; Pierquin et al, 1987; Mazeron et al, 1989; Morrison et al, 1993; Fleming et al, 1995).

Surgical scars were often marked at the initial evaluation but they improved with time. After 4 years, patients were satisfied in 87% of the cases and dermatologists in 79%. This is consistent with the previously published rates of good aesthetic results assessed by patients or physicians, which range from 77% to 93% (Lawrence et al, 1986; Marchac, 1988; Höhmann et al, 1992; Silverman et al, 1992*a*; Bonvallot et al, 1993), and the improvement of the results with time confirms the study of Silverman (Silverman et al, 1992*a*). The good cosmetic result is partly related to almost systematic recourse to frozen section examination, which allows surgeons to spare healthy skin tissue surrounding the tumour. In our trial, the high rate (42%) of additional excisions based on the results of the frozen section examination suggested that surgery was initially as conservative as possible.

Cutaneous necrosis was present in 5% of the patients treated with radiotherapy. This is in agreement with the rates published, which range from 2% to 5% (Nevrkla and Newton, 1974; Brady et al, 1987; Orton, 1978; Pierquin et al, 1987; Crook et al, 1990; Lovett et al, 1990; Lang and Maize, 1991; Wilder et al, 1991). The other major residual abnormalities were, as expected, dyspigmentations and telangiectasia. The rate of good cosmetic results

© Cancer Research Campaign 1997

appears to be lower than those published, but comparison was difficult because of the wide range of published rates of good aesthetic results (60–93%) assessed by patients or physicians (Chahbazian and Brown, 1980; Goldsmith and Sherwin, 1983; Brady et al, 1987; Pierquin et al, 1987; Mazeron et al, 1989; Crook et al, 1990; Lovett et al, 1990; Silverman et al, 1992*b*). The deterioration of the cosmetic appearance with time with radiotherapy confirms the previous results of Silverman (1992*b*) and the small study of Cooper (1988).

This is the first randomized trial of previously untreated BCC comparing surgery and radiotherapy. Surgery proved to be superior to radiotherapy in treatment efficacy and cosmetic result. Thus, for untreated BCC of the face of less than 4 cm, surgery is recommended as first-line treatment and radiotherapy ought to be reserved for patients in whom surgery is contraindicated.

### ACKNOWLEDGEMENTS

We are indebted to C Bognel, MD, C Haie-Meder, MD, and P Opolon, MD, for their contribution to the trial and to Lorna Saint-Ange for her English assistance in writing this manuscript.

### REFERENCES

Ashby MA, Smith J, Ainslie J and McEwan L (1989) Treatment of nonmelanoma skin cancer at a large Australian Center. *Cancer* **63**: 1863–1871

Bonvallot T, Raulo Y, Zeller J, Faivre JM, Horn G and Baruch J (1993) Les carcinomes baso-cellulaires du nez. *Ann Dermatol Venereol* **120**: 209–214

Brady LW, Binnick SA and Fitzpatrick PJ (1987) Skin cancer. In *Principles and Practice of Radiation Oncology*, Perez CA and Brady LW (eds), pp. 377–394. JB Lippincott: Philadelphia

Brown HK and Kempton RA (1994) The application of REML in clinical trials. *Stat Med* **13**: 1601–1617

Chahbazian CM and Brown GS (1980) Radiation therapy for carcinoma of the skin of the face and neck. *JAMA* **244**: 1135–1137

Coebergh JWW, Neumann HAM, Vrints LW, Van Der Heijden L, Meijer WJ and Verhagen-Teulings MT (1991) Trends in the incidence of non-melanoma skin cancer in the SE Netherlands 1975–1988, a registry-based study. *Br J Dermatol* **125**: 353–359

Committee on Guidelines of Care and Task Force on Basal Cell Carcinoma (1992) Guidelines of care for basal cell carcinoma. *J Am Acad Dermatol* **26**: 117–120

Cooper JS (1988) Patients' perceptions of their cosmetic appearance more than ten years after radiotherapy for basal cell carcinoma. *Radiat Med* **6**: 285–288

Cox DR (1972) Regression models and life tables. *JR Stat Soc* **34**: 187–220

Crook JM, Mazeron JJ, Marinello G, Raynal M, Huart J, Leung S, LeBourgeois JP and Pierquin B (1990) Interstitial iridium 192 for cutaneous carcinoma of external nose. *Int J Radiat Oncol Biol Phys* **18**: 243–248

Daly NJ, De Lafontan B and Combes PF (1984) Results of the treatment of 165 lid carcinomas by Iridium wire implant. *Int J Radiat Oncol Biol Phys* **10**: 455–459

Dubin N and Kopf AW (1983) Multivariate risk score for recurrence of cutaneous basal cell carcinomas. *Arch Dermatol* **119**: 373–377

Dutreix A and Marinello G (1987) Source localization and dose calculation, methods. The Paris system. In *Modern Brachytherapy*, Pierquin B, Wilson JF and Chassagne D (eds), pp. 17–42. Masson: New York

Fears TR and Scotto J (1982) Changes in skin cancer morbidity between 1971–1972 and 1977–1978. *J Natl Cancer Inst* **69**: 365–370

Fitzpatrick PJ, Thompson GA, Easterbrook WM, Gallie BL and Payne DG (1984) Basal and squamous cell carcinoma of the eyelids and their treatment by radiotherapy. *Int J Radiat Oncol Biol Phys* **10**: 449–454

Fleming ID, Amonette R, Monaghan T and Fleming MD (1995) Principles of management of basal and squamous cell carcinoma of the skin. *Cancer* **75**: 699–704

Gallagher RP, Ma B, McLean D, Yang CP, HO V, Carruthers JA and Warshawski LM (1990) Trends in basal cell carcinoma, squamous cell carcinoma, and melanoma of the skin from 1973 through 1987. *J Am Acad Dermatol* **23**: 413–421

Goldberg LH (1996) Basal cell carcinoma. *Lancet* **347**: 663–667

Goldsmith H and Sherwin WK (1983) Office radiotherapy of cutaneous carcinomas. Indications in specific anatomic regions. *J Dermatol Surg Oncol* **9**: 47–76

Höhmann DH, Milewski C, Krieger U and Roede J (1992) Asthetische aspekte zur exzision der basaliome im kopf- und halsbereich. *Laryngorhinootologie* **71**: 311–314

Hruza GJ (1994) Mohs micrographic surgery local recurrences. *J Dermatol Surg Oncol* **20**: 573–577

Kaplan ES and Meier P (1958) Non-parametric estimation from incomplete observation. *J Am Stat Assoc* **53**: 457–480

Ko CB, Walton S, Keczkes K, Bury HPR and Nicholson C (1994) The emerging epidemic of skin cancer. *Br J Dermatol* **130**: 269–272

Koch GG, Landis JR, Freeman JL, Freeman DH and Lehnen RG (1977) A general methodology for the analysis of experiments with repeated measurement of categorical data. *Biometrics* **33**: 133–158

Lang PG and Maize JC (1991) Basal cell carcinoma. In *Cancer of the Skin*, Friedman RJ, Rigel DS, Kopf AW, Harris MN and Baker D (eds), pp. 35–73. WB Saunders: Philadelphia

Lawrence CM, Comaish JS and Dahl MGC (1986) Excision of skin tumours without wound closure. *Br J Dermatol* **115**: 563–571

Lawrence CM. Mohs surgery of basal cell carcinoma (1993) A critical review. *Br J Plast Surg* **46**: 599–606

Levi F, La Vecchia C, Te VC and Mezzanotte G (1988) Descriptive epidemiology of skin cancer in the Swiss canton of Vaud. *Int J Cancer* **42**: 811–816

Liu FF, Maki E, Warde P, Payne D and Fitzpatrick P (1991) A management approach to incompletely excised basal cell carcinomas of skin. *Int J Radiat Oncol Biol Phys* **20**: 423–428

Lovett RD, Perez CA, Shapiro SJ and Garcia DM (1990) External irradiation of epithelial skin cancer. *Int J Radiat Oncol Biol Phys* **19**: 235–242

Marchac D (1988) Analysis of a series of 225 cases. In *Surgery of Basal Cell Carcinoma of the Face*, Marchac D (ed), pp. 85–96. Springer: Berlin

Marchac D and Duport G (1980) Résultats carcinologiques et esthétiques du traitement de 138 épithéliomas baso-cellulaires. *Ann Chir Plast* **25**: 127–134

Mazeron JJ, Chassagne D, Crook J, Bachelot F, Brochet F, Brune D, Brunin F, Bunescu U, Daly N, Danczak S, Dubois JB, Grangean M, Hoffstetter S, Koechlin M, Huart J, Labib A, Madelain M, Reynaud-Bougnoux A, Rozan R and Serpantie P (1989) Radiation therapy of carcinomas of the skin of nose and nasal vestibule, a report of 1676 cases by the Groupe Europeen de Curiethérapie. *Radiother Oncol* **13**: 165–173

Morrison WH, Wong PF and Peters LJ (1993) Radiotherapy for basal and squamous cell skin carcinomas. *Cancer Bulletin* **45**: 256–260

Nevrkla E and Newton KA (1974) A survey of the treatment of 200 cases of basal cell carcinoma (1959–1966 inclusive). *Br J Dermatol* **91**: 429–433

Orton CI (1978) The treatment of basal cell carcinoma by radiotherapy. *Clin Oncol* **4**: 317–322

Peto R, Pike MC, Armitage P, Breslow NE, Cox DR, Howard SV, Mantel N, McPherson K, Peto J and Smith PG (1977) Design and analysis of randomized clinical trials requiring prolonged observation of each patient. *Br J Cancer* **35**: 1–35

Petrovich Z, Parker RG, Luxton G, Kuisk H and Jepson J (1987) Carcinoma of the lip and selected sites of head and neck skin. A clinical study of 896 patients. *Radiother Oncol* **8**: 11–17

Pierquin B, Wilson JF, Chassagne D and Mazeron JJ (1987) Skin. In *Modern Brachytherapy*, Pierquin B, Wilson JF and Chassagne D (eds), pp. 273–285. Masson: New York

Preston DS and Stern RS (1992) Nonmelanoma cancers of the skin. *N Engl J Med* **327**: 1649–1662

Reymann F (1980) Basal cell carcinoma of the skin. Recurrence rate after different types of treatment. A review. *Dermatologica* **161**: 217–226

Richmond JD and Davie RM (1987) The significance of incomplete excision in patients with basal cell carcinoma. *Br J Plast Surg* **40**: 63–67

Robins P (1981) Chemosurgery, my 15 years experience. *J Dermatol Surg Oncol* **7**: 779–789

Robins P, Rodriguez-Sains R, Rabinovitz H and Rigel D (1985) Mohs surgery for periocular basal cell carcinomas. *J Dermatol Surg Oncol* **11**: 1203–1207

Roenigk RK, Ratz JL, Bailin PL and Wheeland RG (1986) Trends in the presentation and treatment of basal cell carcinomas. *J Dermatol Surg Oncol* **12**: 860–865

Rothman KJ (1978) Estimation of confidence limits for cumulative probability of survival in life table analysis. *J Chron Dis* **31**: 557–560

Rowe DE, Carroll RJ and Calvin LD (1989) Long-term recurrence rates in previously untreated basal cell carcinoma, implications for patient follow-up. *J Dermatol Surg Oncol* **15**: 315–328

Silverman MK, Kopf AW, Grin CM, Bart RS and Levenstein MJ (1991) Recurrence rates of treated basal cell carcinomas. *J Dermatol Surg Oncol* **17**: 713–718

© Cancer Research Campaign 1997

Silverman MK, Kopf AW, Bart RS, Grin CM and Levenstein MJ (1992*a*) Recurrence rates of treated basal cell carcinomas, Surgical excision. *J Dermatol Surg Oncol* **18**: 471–476

Silverman MK, Kopf AW, Gladstein AH, Bart RS, Grin CM and Levenstein MJ (1992*b*) Recurrence rates of treated basal cell carcinomas, X-ray therapy. *J Dermatol Surg Oncol* **18**: 549–554

Tromovitch TA and Stegman SJ (1978) Microscopic-controlled excision of cutaneous tumors. Chemosurgery, fresh tissue technique. *Cancer* **41**: 653–658

Wilder RB, Kittelson JM and Shimm DS (1991) Basal cell carcinoma treated with radiation therapy. *Cancer* **68**: 2134–2137

*© Cancer Research Campaign 1997*